# Benchmarking Static Analysis for PHP Applications Security

**DOI:** 10.3390/e27090926

**Published:** 2025-09-03

**Authors:** Jiazhen Zhao, Kailong Zhu, Canju Lu, Jun Zhao, Yuliang Lu

**Affiliations:** 1 College of Electronic Engineering, National University of Defense Technology, Hefei 230037, China; jiazhenzhao@nudt.edu.cn (J.Z.); lucanju17@nudt.edu.cn (C.L.); zhaojun17@nudt.edu.cn (J.Z.); 2 Anhui Province Key Laboratory of Cyberspace Security Situation Awareness and Evaluation, Hefei 230037, China

**Keywords:** benchmarking, static analysis, php application security, assessment system, information entropy, uncertainty quantification

## Abstract

PHP is the most widely used server-side programming language, but it remains highly susceptible to diverse classes of vulnerabilities. Static Application Security Testing (SAST) tools are commonly adopted for vulnerability detection; however, their evaluation lacks systematic criteria capable of quantifying information loss and uncertainty in analysis. Existing approaches, often based on small real-world case sets or heuristic sampling, fail to control experimental entropy within test cases. This uncontrolled variability makes it difficult to measure the information gain provided by different tools and to accurately differentiate their performance under varying levels of structural and semantic complexity. In this paper, we have developed a systematic evaluation framework for PHP SAST tools, designed to provide accurate and comprehensive assessments of their vulnerability detection capabilities. The framework explicitly isolates key factors influencing data flow analysis, enabling evaluation over four progressive dimensions with controlled information diversity. Using a benchmark instance, we validate the framework’s feasibility and show how it reduces evaluation entropy, enabling the more reliable measurement of detection capabilities. Our results highlight the framework’s ability to reveal the limitations in current SAST tools, offering actionable insights for their future improvement.

## 1. Introduction

Web applications are the internet’s primary way of sharing information and services. PHP is recognized as the most popular server-side language, used by 73.6% of websites [1]. Prominent platforms like Tesla’s official website and Facebook rely on PHP due to its versatility and efficiency. PHP offers two significant advantages. Firstly, as a weakly typed language, it features dynamic capabilities and a vast array of built-in functions, making it both user-friendly and easy to learn. Secondly, being an interpreted language specifically designed for web application development, PHP provides robust support for handling HTTP requests, processing form data, and generating HTML content. Additionally, it integrates seamlessly with popular web servers like Apache and Nginx. However, the widespread adoption of PHP applications also brings significant security concerns. Vulnerabilities in PHP applications, if maliciously exploited, can lead to data breaches, service interruptions, and severe security issues. These threats pose substantial risks to personal privacy, business operations, and even national security.

Faced with the threats posed by PHP applications, many methods have been proposed to detect vulnerabilities in PHP applications, among which static analysis is an important method that can cover 100% of the code [2] and detect potential problems in the early stages of program development. In fact, some studies suggest that static application security testing (SAST) tools can detect approximately half of existing security vulnerabilities [3].

After an in-depth analysis of static vulnerability analysis solutions for current PHP applications, we found that the lack of robust benchmarks is a key factor limiting the effectiveness improvement of these solutions. The existing evaluation methods usually rely on selecting some real-world PHP applications as experimental datasets to evaluate the methods’ vulnerability detection ability, false positive rate, and performance consumption. However, such methods introduce uncontrolled evaluation entropy [4] by failing to standardize or quantify the inherent structural and semantic variability among different applications. This uncontrolled information diversity makes it difficult to isolate key variables, obscures the true information gain achieved by different tools, and complicates the explanation of why certain static analysis methods perform well on some applications while underperforming on others [5,6,7].

The currently available benchmarks for PHP SAST tools are relatively limited. To overcome this challenge, researchers have worked hard to identify the key elements of potential vulnerability test cases in PHP applications and attempted to generate comprehensive test cases by traversing and combining these elements [8,9]. This approach led to two benchmarks included in the Software Assurance Metrics and Tool Evaluation (SAMATE) Project from NIST: *PHP Vulnerability Test Suite* [10] and *PHP Test Suite* [11]. However, there are two main issues with the generation method of this benchmark.

First, there is insufficient control over experimental variables, which leads to high evaluation entropy. The combinatorial generation of multiple factors makes it difficult to quantify the information contribution of each factor, increasing uncertainty and obscuring the causal relationships behind the failure of SAST tools [12,13,14]. Second, the generated test cases are often too small in scale, while they provide a broad coverage of elemental patterns, they exhibit low information diversity and lack the structural and semantic complexity of real-world applications. As a result, they fail to capture the entropy distribution present in practical applications, which leads to a mismatch between the tool’s performance on small test cases and its ability to handle vulnerabilities in real applications [15].

Our goal is to address the limitations of existing work and propose a comprehensive benchmark design approach for evaluating SAST tools against PHP application vulnerabilities. This method aims to systematically assess the detection performance of PHP SAST tools while quantifying the uncertainty inherent in static analysis, thereby providing clear guidance for improving their effectiveness. For this purpose, we conducted in-depth research, including an analysis of the taint-type vulnerabilities in popular PHP applications, as well as a comprehensive analysis of the state-of-the-art SAST tools. We yielded three key findings. First, in PHP applications, taint-type vulnerabilities rarely manifest within a single execution path but exhibit high information entropy due to complex, interprocedural propagation. Second, the data flow analysis in current SAST tools suffers from entropy accumulation. Although many claim to support interprocedural analysis, we observed uncertainty amplification when propagating taint information across function boundaries, and their primarily flow-sensitive analyses struggle to reduce entropy in the presence of extensive branching. Third, PHP applications make extensive use of dynamic features, which significantly increase structural and semantic entropy. These features create unpredictable execution paths, further challenging SAST tools to maintain precise vulnerability detection [16,17,18,19].

Leveraging this observation, we propose a comprehensive, entropy-aware benchmark design approach for evaluating SAST tools that detect PHP application vulnerabilities. This approach performs an incremental assessment of tool capabilities while explicitly modeling and controlling evaluation entropy, which reflects the uncertainty and information diversity present in static analysis, across four progressive dimensions: identifying the three elements of taint analysis, basic data flow analysis, complex semantic analysis, and identifying vulnerabilities in real-world applications. The first dimension evaluates the coverage of SAST tools on taint sources, sanitization, and sinks, which form the foundation for accurately detecting taint-type vulnerabilities and measuring the entropy reduction achieved when mapping input data to potential exploit paths. The second dimension assesses flow-sensitive, context-sensitive, and interprocedural analysis capabilities, which are critical because insufficient propagation modeling amplifies uncertainty and increases entropy across data flow paths in complex PHP applications. The third focuses on the tools’ ability to analyze challenges such as type inference, built-in functions, and dynamic features, unique characteristics of the PHP language that introduce significant structural and semantic entropy and require precise constraint modeling to avoid uncertainty explosion. Finally, the benchmark evaluates the effectiveness of SAST tools in handling real-world applications, where long data flows and deeply nested contexts generate high-entropy vulnerability patterns that test the tools’ ability to deal with uncertainty and extract actionable information.

In summary, this paper makes the following contributions:

- We propose a systematic, entropy-aware evaluation framework for PHP SAST tools. It progressively assesses vulnerability detection capabilities across four dimensions: identifying the three elements of taint analysis, basic data flow analysis, complex semantic analysis in PHP applications, and detecting vulnerabilities in real-world applications. By explicitly modeling and controlling evaluation entropy, the framework reduces uncertainty and balances information diversity throughout the assessment.
- Our method is the first to isolate and differentiate critical factors affecting data flow analysis, providing a fine-grained assessment of SAST tools’ foundational analytical capabilities. By pinpointing where entropy accumulates in the analysis pipeline, it uncovers inherent weaknesses and offers clear guidance for optimizing tool performance.
- We demonstrate the feasibility of this framework through a dedicated benchmarking instance, use it to evaluate seven popular SAST tools, and reveal key limitations in their current designs. The benchmark instance is publicly available at https://github.com/xjzzzxx/PSAbench to facilitate future research and reproducibility.

## 2. Related Work

Although there have been numerous studies on PHP SAST methods in recent years, there is relatively little benchmarking work for PHP SAST, and existing evaluations lack mechanisms to quantify analysis uncertainty or control evaluation entropy.

There are two existing benchmarks for PHP SAST tools, *PHP Vulnerability Test Suite* [10] and *PHP Test Suite* [11], both adopt a modular design concept and build test cases by flexibly combining diverse source code snippets, achieving large-scale automated generation of test cases. However, these generated test cases often fail to fully reflect the characteristics of real-world applications in terms of complexity and code structure, making it difficult to fully represent the analytical challenges in practical applications. In addition, since test cases are constructed through the combination of multiple modules, the resulting evaluation exhibits high entropy, with mixed analytical challenges that increase uncertainty and make it difficult to isolate the contribution of individual factors, making it difficult for us to accurately determine which specific modules SAST tools perform poorly based on the analysis results. This limitation not only hinders our in-depth analysis of SAST tool evaluation results but also limits our effective exploration of tool performance improvement strategies.

Nunes et al. [2] proposed a benchmark testing method for static analysis tools, which improves the relevance and accuracy of evaluation results by considering different ranking metrics and adjusting workloads based on scenario characteristics, and constructed a benchmark testing dataset composed of WordPress plugins. However, the entropy distribution of such a dataset is narrow due to limited functional diversity and structural complexity, which constrains its ability to reflect the information variability present in large, real-world applications. However, the benchmark tests composed of WordPress plugins cannot fully represent web applications in the real world. The main limitations include single-functionality modules lacking coverage of multi-functional, integrated applications; fixed architectures that fail to reflect the diversity of technology stacks and frameworks; insufficient dynamic features, making it difficult to capture complex runtime behaviors; limited security scenarios, excluding a broad range of threat models; and smaller codebases with lower complexity, which are inadequate for evaluating tool performance in large, complex systems.

Al Kassar et al. [18] explored the impact of complex code patterns in web applications on the analytical capabilities of SAST tools. They found that these code patterns are not only very common in PHP applications, but also proved through code conversion that these code patterns are blind spots in the analysis capabilities of SAST tools. However, these patterns often include multiple complex factors that increase analysis entropy and amplify uncertainty propagation, making it difficult for researchers to identify which specific variable dominates tool failures, such as intricate data flows, taint propagation through built-in functions, or extensive use of object-oriented programming. This “many causes, one effect” situation makes it difficult for researchers to pinpoint the exact reasons for SAST tool analysis failures, as multiple factors may independently or collectively contribute to the inability to handle test cases correctly.

In PHP SAST research, various studies typically claim to have developed new analytical capabilities. For instance, PhpSafe [20] claims to analyze object-oriented web applications, RIPS [21] asserts a high degree of emulation for PHP built-in functions, and TCHECKER [22] claims precise interprocedural analysis capabilities. However, the evaluation of these methods is often limited to real-world applications selected by the researchers. This evaluation approach informs readers about the methods’ ability to detect vulnerabilities but fails to assess their claimed analytical capabilities or quantify the entropy reduction achieved by these new features, making it difficult to measure whether they truly reduce uncertainty in vulnerability detection. For professional web application security researchers, verifying these claims by delving into the tools’ source code can be highly time-consuming. In the absence of unit tests, accurately evaluating the tools’ claimed new capabilities becomes even more challenging, let alone enabling ordinary users to easily verify these claims.

Our previous research [23] focused on developing a more efficient static application security testing (SAST) method for PHP applications. During the design of its experimental evaluation, however, we recognized that there is currently a lack of a comprehensive and systematic evaluation system for PHP SAST tools, one that explicitly models evaluation entropy and controls information diversity, which are essential for objective capability assessment. This realization forms the starting point of the present study. This paper aims to explore in depth how to effectively and comprehensively evaluate the capabilities of existing SAST tools. To this end, we construct a targeted capability evaluation framework that deconstructs the essential capabilities of static analysis into incrementally layered factors. The framework is designed to progressively reduce evaluation entropy by isolating layered analytical factors and measuring their contribution to uncertainty reduction in vulnerability detection. With this benchmark dataset, we can objectively assess the current functionality of SAST tools and provide guidance for future improvements.

## 3. Background and Motivation

### 3.1. Static Analysis

Static analysis is a method used to analyze program semantics in order to identify potential errors and vulnerabilities without actually running the code. PHP applications often provide extensive functionality and are highly dependent on user input. When this input data is insufficiently sanitized and used in dangerous operations, such as data output, database queries, or command execution, it can lead to vulnerabilities. These vulnerabilities, referred to as taint-style vulnerabilities [22], occur when three specific conditions are satisfied. First, the program must receive user input, referred to as a taint source. Second, the taint propagates within the program without being adequately sanitized (sanitization). Finally, the taint is used in dangerous operations (sink).

Therefore, taint analysis in static analysis is the most commonly used method for detecting vulnerabilities in PHP applications. Taint analysis focuses on three key elements: taint source, sanitization, and sink. Correct identification of these elements ensures accurate modeling of taint-style vulnerabilities. Additionally, taint analysis must track the taint flow, requiring basic data flow analysis capabilities, such as interprocedural analysis to track taint propagation between processes. Furthermore, the dynamic nature of PHP applications, challenges related to type inference, and the complexities introduced by built-in functions demand that taint analysis handle these issues effectively [18,22], while current taint analysis methods have shown some success, the lack of a standardized benchmark dataset makes it difficult to accurately evaluate their limitations and identify specific areas for improvement. As a result, these methods often suffer from high false positive and false negative rates, limiting their practicality in real-world production environments [24]. In particular, uncertainty in taint propagation can gradually increase across function boundaries and branching structures, a phenomenon we refer to as entropy accumulation, where analysis imprecision compounds along longer execution paths.

### 3.2. Benchmark

The benchmark for PHP application static analysis is the standard used to assess and compare the effectiveness of these methods or tools, typically consisting of three main components [25]:

- Metrics: lighthouse in the benchmark, guiding the assessment and improvement of tool performance with clear, quantifiable indicators that illuminate key performance and help navigate potential performance bottlenecks.
- Workload: a representative set of test cases that can simulate workloads in actual usage scenarios to evaluate the performance of tools in real-world applications, providing a foundation for metrics evaluation.
- Procedure: the step-by-step guide ensures consistent testing. It outlines methodologies for preparing workloads, executing tests, and analyzing metrics, guaranteeing the reliability of benchmarking.

However, there is currently no consensus on metrics. In previous studies, researchers identified three test case characteristics required to calculate the following metrics [15,26]: **(A) Statistical significance**: test cases must have a certain scale that can expose the diversity of defects to generate statistical significance. **(B) Ground truth**: we need to know the location of all defects in the test cases to make it easier to evaluate tools’ alarms. **(C) Relevance**: test cases must represent real-world software used in production environments or developed according to industry standards. However, there are no test cases that simultaneously meet these three characteristics, as it requires a massive amount of work to create such test cases, and it is also necessary to ensure that certain new features can be added to the test cases in a timely manner according to the update time of the development language. We can find test cases that combine two of these characteristics: real-world production applications satisfy A and C, applications containing known vulnerabilities satisfy C and part B (because it can never be determined whether there are undiscovered vulnerabilities in an application), and small test cases satisfy A and B.

### 3.3. Motivation

Two existing PHP SAST tools benchmark, *Vulnerability Test Suite* [10] and *PHP Test Suite* [11], are composed of many small test cases, which can only satisfy the statistical significance (A) and ground truth (B) among the three test case characteristics. However, in addition to lacking relevance (C), these test cases also suffer from insufficient control of experimental variables, which weakens the benchmarks’ ability to guide the assessment and improvement of tools’ performance. Listing 1 shows a test case inspired by *PHP Vulnerability Test Suite*. On line 4, user-controllable dangerous input is accepted through the $_GET variable, and the taint is passed to $array[1] and subsequently to $tainted on line 6. Afterwards, on line 9, $tainted is processed by the built-in function (in_array). Depending on the Boolean value returned by the function, the program determines whether to execute the if-branch or the else-branch. If the value of $tainted is not equal to safe1 or safe2, the program executes the if-branch. In this case, the value of $tainted remains unchanged and still carries the taint. Finally, on line 15, the taint is passed to the sink function(echo), resulting in an XSS vulnerability.

**Listing 1.** Example of a test case.

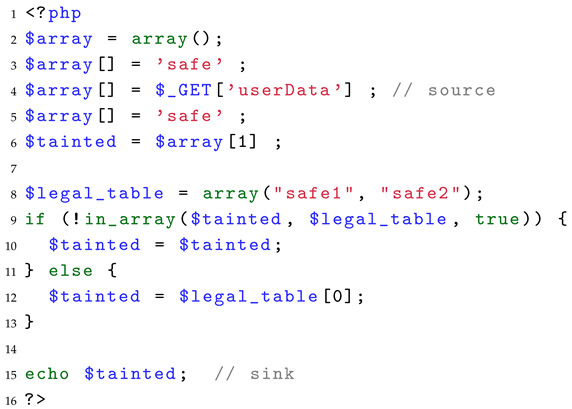



The state-of-the-art PHP SAST tool Tchecker [22] and RIPS [21] could not detect the vulnerability in this test case. Still, it is difficult to determine what factors caused their detection failure because there is more than one analysis challenge in this test case, such as the if-else branch on lines 9–12 requiring path-sensitive analysis capabilities, the semantic modeling of the built-in function in_array on line 9, and the analysis of taint propagation in reading and writing of $array elements.

In summary, we derive two key insights. First, the test cases in the existing PHP SAST tool benchmarks exhibit high evaluation entropy because multiple analytical challenges are mixed together, making it difficult to isolate the contribution of individual factors and limiting their ability to guide tool evaluation and improvement. Second, both benchmarks, based on small test cases and those built from real-world applications, cover only part of the information diversity needed for a comprehensive assessment, resulting in an incomplete entropy distribution across test cases.

Therefore, our intuition is that it is feasible to combine the advantages of these two benchmark types and construct a benchmark that approximates the full entropy spectrum by satisfying the characteristics of the three test cases as much as possible. Moreover, to enhance the benchmark’s effectiveness in evaluating and improving tool performance, the entropy contributed by different analytical challenges should be decoupled as much as possible in small test cases rather than being mixed, enabling controlled uncertainty and more precise performance attribution.

## 4. Methods

As we explained in the previous section, our goal is to combine the advantages of these two types of benchmarks and separate the analysis challenges in a way that reduces evaluation entropy and enables more precise attribution of tool performance.

For the mixed problem of analysis challenges in small test cases, we address it by leveraging the technical characteristics of PHP application taint analysis. Firstly, the three key elements of taint analysis taint source, sanitization, and sink, form the primary information channels for detecting taint type vulnerabilities. Evaluating the ability of SAST tools to correctly identify these elements provides a measure of entropy reduction when mapping input data to potential exploit paths. Secondly, the dynamic pages and navigation complexity [27] of PHP applications introduce significant branching and long execution paths, which increase analysis uncertainty and amplify entropy across data flow propagation. Therefore, we evaluate whether SAST tools can perform accurate flow-sensitive, context-sensitive, and interprocedural analysis to control this uncertainty and prevent entropy accumulation during source-to-sink taint propagation. Thirdly, challenges such as type inference, built-in functions, and dynamic features introduce high structural and semantic entropy unique to PHP. Failure to model these correctly results in premature termination of data flow analysis and uncontrolled uncertainty, directly impacting the effectiveness of vulnerability detection. By decoupling the current analysis challenges into these three categories, we construct an entropy-aware assessment that characterizes the fundamental detection capabilities of SAST tools in PHP applications.

Our intuition is that SAST tools capable of correctly addressing these three entropy-contributing dimensions may still face difficulty detecting vulnerabilities in real-world applications due to the inherently higher information diversity and uncertainty in long data flows and nested contexts. However, tools that cannot reduce entropy and control uncertainty in these three areas are certain to fail in real-world scenarios because these abilities are prerequisites for handling the high-entropy execution space of PHP applications. Due to the limited Lines of Code (LoC) in the small test cases, they inevitably have lower entropy distribution and cannot fulfill the relevance (C). By combining real-world applications, we aim to create a comprehensive benchmark that balances and spans the full entropy spectrum, meeting the three key characteristics of test cases.

For real-world applications, it is impossible to guarantee the absence of unknown vulnerabilities, and for SAST tools, the ability to detect unknown flaws is itself a measure of handling uncertainty. To ensure a controlled entropy baseline and establish partial Ground Truth (B), we control the version of real-world applications to guarantee a known set of vulnerabilities. Additionally, we avoid artificial vulnerability insertion [28], which would distort the natural entropy distribution and compromise the relevance (C) of the application. Through this benchmark, we evaluate the effectiveness of SAST tools in controlling uncertainty and extracting actionable information from long data flows and high-entropy contexts present in real-world PHP applications.

**Figure 1:**
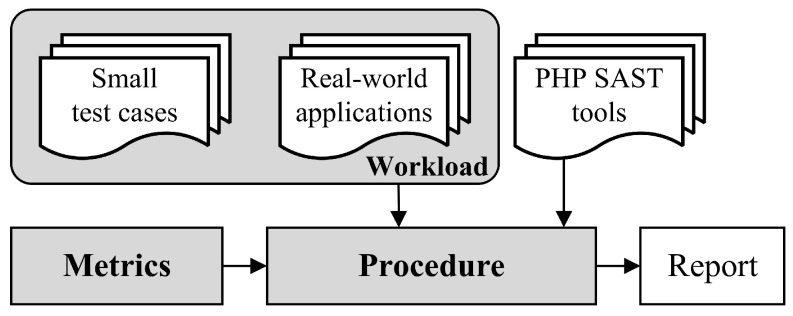
Architecture of the benchmark.

Consequently, the combined benchmark of small test cases and real-world applications provides a progressive, entropy-aware evaluation of PHP SAST tools’ vulnerability detection capabilities across four dimensions. The overall benchmark architecture is illustrated in Figure 1. Our approach is composed of three components that are introduced in the following sections:

### 4.1. Metrics

We evaluate the vulnerability detection capability of the PHP application SAST tool from four dimensions, so we need to propose corresponding metrics for each dimension. Among them, the evaluation of the three elements of taint analysis identification capability, basic data flow analysis capability, and complex semantic analysis capability are all provided by small test cases, with the focus on determining whether they have the capability. Therefore, we selected the false positive rate (FPR) and true positive rate (TPR) as the fundamental evaluation metrics to reflect the tools’ false alarm and detection capabilities.

To further enhance the comprehensiveness of the performance evaluation, we introduced the Benchmark Accuracy Score (BAS) metric [2], calculated as follows:

BAS=(TPR−FPR)×100

We adopted BAS over standard metrics such as accuracy, precision, or F1 score because our test cases are not designed to provide balanced datasets, but to assess whether specific capabilities exist. In such imbalanced settings, accuracy can be dominated by the number of negative cases, while precision and F1 heavily depend on the proportion of positives and negatives, which may lead to misleading interpretations. BAS, on the other hand, directly combines TPR and FPR into a single value, offering a balanced and intuitive measure of how well a tool achieves detection while controlling false alarms. This makes BAS particularly suitable for static analysis evaluation, where both missed detections and excessive false positives are critical concerns. Due to the limited LoC of each small test case, runtime performance metrics such as analysis time are excluded to avoid interference from external factors.

For the capability to detect real software vulnerabilities, in addition to commonly used FPR and TPR, we consider further distinguishing the differences between vulnerabilities detected by various SAST tools, because detecting the same TPR does not mean that the vulnerabilities detected by the tools are exactly the same. Therefore, using unique positive (NP) represents the number of unique vulnerabilities detected by a certain tool but not detected by other tools, and using new vulnerabilities (NV) represents the number of unknown vulnerabilities detected by a certain tool. In addition, considering that analysis time is also an important indicator of the practicality of SAST tools in production environments, we use Time to represent the analysis time of each tool.

### 4.2. Workload

In designing the workload, we follow the principle of a “separation requirement”, which refers to isolating individual analysis challenges so that each test case focuses on a single dimension of difficulty without interference from others. We discuss the setting of Workload from four dimensions.

**Identify the three elements of taint analysis (A1)**: Currently, there is no dataset specifically designed for identifying the three elements of taint analysis. Therefore, we conducted a comprehensive investigation of all built-in PHP [29] functions. Based on the definitions of sources, sanitization, and sinks mentioned in the background section, we analyzed and matched the semantics of these built-in functions to complete the classification and constructed the workload for this part. More specifically, for taint sources, we designate five user-controllable superglobal variables as sources of taint: $GET, $POST, $FILES, $COOKIE, and $REQUEST. For sanitization functions, we define them as functions that disrupt the attack semantics of their parameters with respect to a specific vulnerability type. If the return value processed by such a function can no longer trigger the relevant vulnerability, effectively interrupting meaningful taint propagation, the function is considered a sanitization function. For example, as shown in Listing 2, the sink function is echo, and thus we consider potential XSS vulnerabilities. After being processed by the abs function, the tainted variable $tainted is transformed into a positive integer, which cannot carry the malicious semantics required to trigger an XSS attack (e.g., injection of <script> tags). For sink functions, we selected security-sensitive functions in PHP, such as system, exec, unlink, file_get_contents, mysql_query, move_uploaded_file, and echo. When unprocessed tainted variables are passed into these functions, they may result in vulnerabilities.

**Listing 2.** Example of a sanitization function.

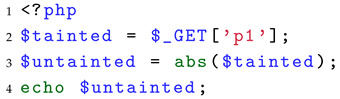



Below, we use specific test cases as examples to illustrate how to evaluate the ability of SAST tools to identify the three key elements of taint analysis.

For the source, we traverse all the source points of PHP applications and use the statement echo as the sink. Listing 3 shows a test case for evaluating the source identification capability. According to the separation requirement, this part should ideally focus solely on source points without involving taint propagation. However, if no propagation is introduced, they cannot be evaluated as effective sources of taint, since their impact would not manifest. Therefore, we slightly relax the separation requirement by constructing a minimal taint propagation process, which enables us to evaluate the capability of SAST tools to correctly recognize source points.

**Listing 3.** Example of a test case of source identification.

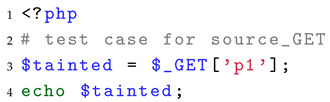



For sanitization, we fix the source as GET and the sink as echo, and then iterate through all sanitization functions in PHP based on this setup. In our previous work [23], we found that there is a class of functions in sanitization that can be used to recover taint through paired functions. We called them reversible sanitization and added them to our benchmark to evaluate whether SAST tools can recognize such sanitization functions. Specifically, this type of function typically involves encoding/decoding or encryption/decryption operations. When a function A encodes or encrypts a string, there exists a corresponding function B that can reverse the operation and restore the encoded or encrypted data. Thus, when a tainted variable is processed by function A, the taint is temporarily sanitized but can be restored when processed by function B. Listing 4 presents two scenarios of reversible sanitization functions. In lines 3–5, only htmlspecialchars is executed, so the taint is sanitized and no vulnerability exists. In lines 7–9, both htmlspecialchars and htmlspecialchars_decode are executed in sequence, restoring the taint in line 9 and resulting in an XSS vulnerability in line 10. For SAST tools, reporting no vulnerability in the first case (lines 3–5) is considered a true positive, while identifying a vulnerability in the second case (lines 7–9) is also considered a true positive.

**Listing 4.** Example of a test case of sanitization identification.

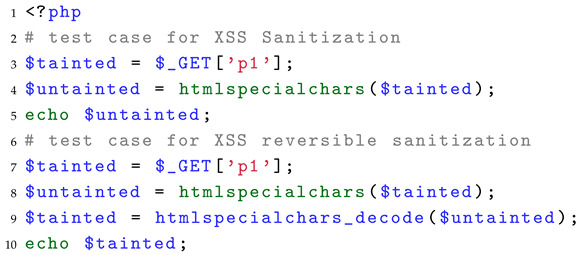



For the sink, we fix the source as GET and do not use sanitization. We iterate through the sink functions in PHP that may cause different vulnerabilities to evaluate the types of vulnerabilities supported by SAST tools for detection. In addition, the sink function may have multiple parameters, and usually, only one parameter carrying a taint can lead to vulnerabilities. For such multi-parameter sink functions, we set up an example that passes a taint to non-hazardous parameters to determine whether the SAST tool has modeled hazardous parameter positions. Listing 5 presents the test case for evaluating the sink identification capability. We use a paired positive and negative test case to assess whether the SAST tool supports the recognition of dangerous parameters in the dangerous function, as illustrated in lines 3 to 8 of the code. When the sink function has only one required parameter and the other parameters are optional, we do not consider it as a multi-parameter sink function in our evaluation, as shown in the exec in lines 9–10 of the code.

**Listing 5.** Example of a test case of sink identification.

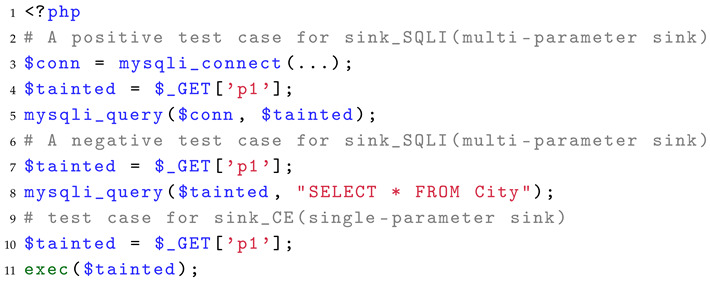



In summary, A1 is designed to assess whether SAST tools can correctly model sources, sanitization functions, and sinks in PHP applications. We conducted a comprehensive survey of all PHP built-in functions and classified them based on the definitions of source, sanitization, and sink. The dataset was constructed step by step, with each component (source, sanitization, and sink) isolated to minimize interference from other factors.

To elaborate, for sources, we created minimal vulnerable code snippets with straightforward taint propagation, such as a two-line example involving an XSS vulnerability. These examples require SAST tools to recognize basic sources like GET and fundamental sinks like echo. For sanitization, we extended these examples by adding sanitization functions, testing whether the tools properly model them. Similarly, sink-related examples fixed the source as GET and excluded any sanitization, focusing on detecting the correct identification of sinks. This approach effectively delineates the boundaries of SAST tools in modeling these essential components.

**Basic data flow analysis capabilities (A2)**: Similarly, there is currently no dataset specifically designed for evaluating the basic capabilities of PHP SAST tools in data flow analysis. Therefore, we conduct in-depth investigations into known vulnerabilities in PHP applications, analyze the taint propagation of these vulnerabilities, and study what capabilities PHP SAST tools need to have to detect these vulnerabilities. We found that these vulnerabilities have longer data flows, more branch judgments, more interprocedural data flows, and more complex contexts compared to small test cases. Therefore, PHP SAST tools need to have flow-sensitive analysis, context-sensitive analysis, and interprocedural analysis capabilities to complete the detection tasks for these vulnerabilities.

**Figure 2:**
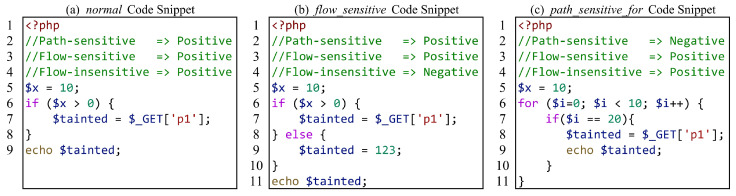
A test case of flow-sensitivity analysis capability evaluation.

For the evaluation of flow-sensitivity analysis capabilities, we consider three levels of abilities: flow-insensitive, flow-sensitive, and path-sensitive analysis capabilities. Figure 2 shows a sample code of test cases for evaluating the flow-sensitivity analysis capabilities. Overall, we cleverly designed the code execution logic of the test cases, and through the combined verification of two or more test cases, we can determine the analytical ability of the PHP SAST tool. As shown in the figure, the code in Figure 2a is a simple if-branch, so whether it is the flow-insensitive method without considering branch logic or the flow-sensitive method, the analysis of this sample code can be performed in order from top to bottom. Figure 2b introduces the else-branch and inserts an assignment statement below the if-branch to sanitize the tainted variable $tainted. If it is a flow-insensitive method and still parsed in top-to-bottom order, the result will be that the $tainted variable does not carry any taint, while the flow-sensitive method does not. In Figure 2c, a for-branch is added so that the program only accepts the taint and triggers the sink when the value of $i is equal to 20. For flow-sensitive, it ignores conditional predicates to track and record the situation of all branches, so it assumes that an if-branch may also be executed, leading to a false positive report.

**Figure 3:**
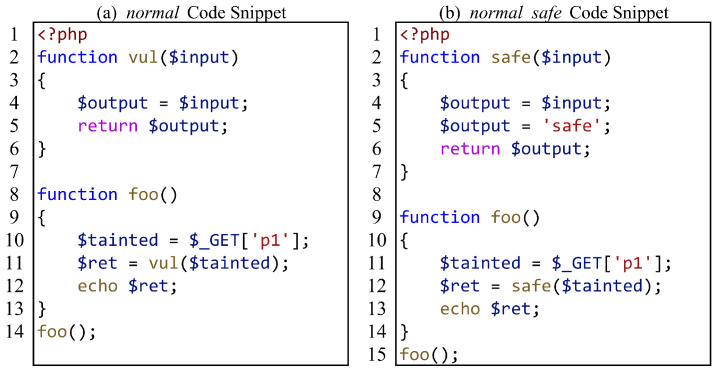
A common function call test case.

For the evaluation of interprocedural analysis capabilities, we consider two scenarios: function calls (including method calls) and file inclusion. For function calls, we focus on whether SAST tools can correctly analyze the data flow of function call edges and function return edges. Figure 3 shows a function call test case, where (a) has a vulnerability and (b) is the secure version of (a). In Figure 3a, the taint is passed as a parameter into the function vul, propagates within the function, and is then returned through a return statement. On line 11, the variable $ret receives the tainted value and triggers the sink on line 12. Figure 3b adds an assignment to the safe function to sanitize the taint. By combining these two test cases for verification, we can determine whether the SAST tool can handle the most common function call scenarios.

**Figure 4:**
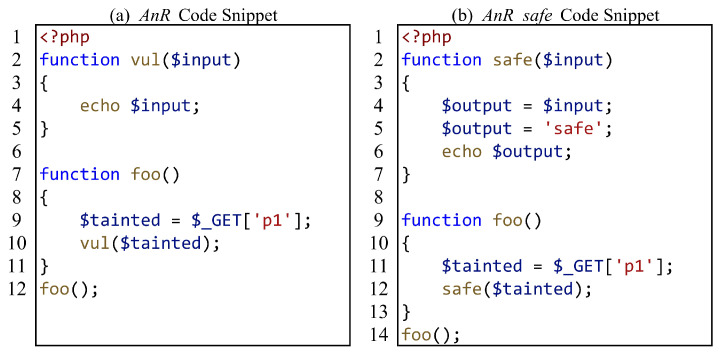
An Arg but not Ret(AnR) function call test case.

On the other hand, because SAST tools often make assumptions, taking Figure 3a as an example, assuming that the function vul on line 11 is a function that can propagate taint, and then directly assuming that the return value $ret of the function also carries taint because the parameter $tainted of the vul function carries taint. That is to say, the SAST tool did not conduct interprocedural analysis, and the vulnerability of the test case was only determined based on the assumption of success. Therefore, we separate the data flows of function call edges and function return edges to further evaluate the function call analysis capability of SAST tools. Figure 4 shows a test case where function parameters are passed, but function return values are not used. Compared with Figure 3, the sink is inside the called function and no longer triggers the sink by returning a tainted variable. Therefore, it can be determined whether the SAST tool accurately analyzes the data flow on the edge of the function call.

**Figure 5:**
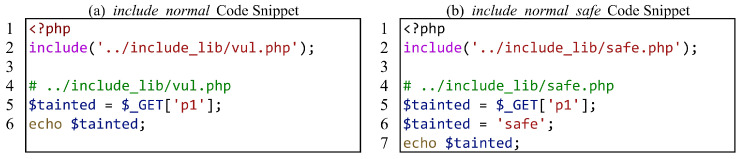
A common file inclusion test case.

For file inclusion, we focus on whether SAST tools can correctly analyze the data flow within the included files. Figure 5 shows a test case, where (a) has vulnerabilities and (b) is the secure version of (a). It is worth mentioning that due to inconsistent vulnerability report samples generated by various SAST tools, some tools only report the file where the sink is located and do not report the propagation process from source to sink. When the main file and the included files are in the same directory, we use some SAST tools to analyze the directory, and the report only shows that there are vulnerabilities in the included files, which makes it impossible for us to evaluate whether these SAST tools can analyze the file content. Therefore, we cleverly separate the main file and the included files into two directories and use SAST tools to analyze the directory where the main file is located, to make the correct assessment.

**Figure 6:**
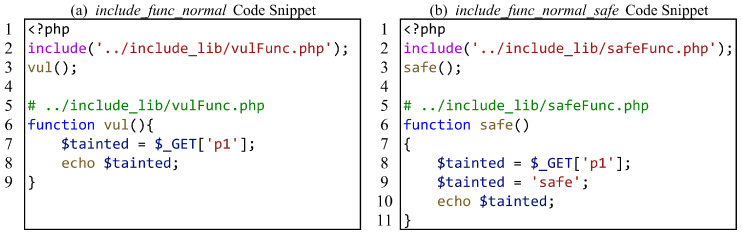
Another file inclusion test case.

Calling functions defined in the included file is another scenario for file inclusion interprocedural analysis. Figure 6 shows the test case for calling functions defined in the included file. Similarly, we still adhere to the strategy of separating the main file from the directory containing the included files.

For the evaluation of context-sensitive analysis capability, we also use the joint validation of two test cases to determine whether the SAST tool has context-sensitive analysis capability, mainly evaluating the context-sensitive analysis capability in two scenarios: function call and method call. Because context-insensitive analysis typically involves function summarization of the called function, in this capability assessment, two function call points are set, one causing a vulnerability and the other not. Listing 6 shows a test case in a function call scenario. When the SAST tool reports a vulnerability, it indicates that the tool has context-sensitive analysis capabilities in that scenario. When the SAST tool reports no vulnerabilities or two vulnerabilities, it indicates that the tool only has context-insensitive analysis capabilities.

**Listing 6.** A test case of context-sensitive assessment.

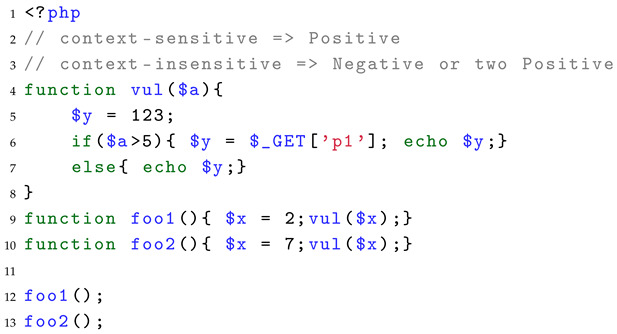



To summarize, A2 focuses on evaluating the data flow analysis capabilities of SAST tools, including flow-insensitive, flow-sensitive, path-sensitive, interprocedural, and context-sensitive analyses. To control experimental variables, we fixed GET as the source, echo as the sink, and excluded sanitization functions. This standardization ensures that the evaluation isolates the tools’ abilities to handle different levels of data flow analysis without interference.

**Complex semantic analysis capabilities (A3)**: For a long time, PHP’s SAST tools have faced complex challenges in type inference, dynamic features, and built-in functions during the analysis process. Accurate type inference is the foundation for solving dynamic features analysis, function/method call addressing, and other analysis scenarios. Only by correctly inferring the types of relevant variables can the subsequent analysis be carried out correctly. The behavior of dynamic features may only be determined at runtime, and static analysis requires understanding dynamic features without executing code, which is a challenging task. The built-in functions of PHP are implemented in C language, and for SAST tools, the internal implementation of these built-in functions is a black box, making it difficult to analyze their internal data flow. In previous studies [18], researchers delved into the impact of these complex semantics on SAST tools and made the dataset open-source. Based on their work, we reclassified their dataset according to type inference, dynamic features, and built-in functions, and corrected some erroneous test cases in the dataset. It is worth mentioning that because test cases with complex semantics usually involve branch structures and function calls, it is difficult to control experimental variables well. However, we believe that SAST, which has achieved excellent results in the evaluation of the three elements of taint analysis recognition capability and the evaluation of basic data flow analysis capability, will not be troubled in these aspects and can effectively evaluate SAST’s ability in complex semantic analysis. This is also the core advantage of our proposed SAST capability progressive assessment method. This method gradually increases the difficulty of analysis through a carefully designed hierarchical structure, with each level built on the foundation of the previous level, ensuring that the depth and breadth of analysis continue to increase as the levels progress.

**Real-world application vulnerability detection capability (A4)**: Evaluating the capability of SAST tools to detect real-world application vulnerabilities is the most essential requirement for assessing the capability of SAST tools. For the selection of real-world applications, we have two main principles: first, the application must be widely used, and second, the latest version of the application must be released within ten years. The first principle ensures that the applications we select have universality and can represent applications developed according to industry standards as much as possible, as this often better represents the current trends and development trends in the production environment than applications used by a few people. The second principle ensures that the PHP version used by the application is not too outdated, avoiding vulnerabilities that are outdated or no longer relevant. The real-world applications selected based on such principles can effectively assess the capability of SAST tools in detecting vulnerabilities in real-world applications.

More specifically, we selected 24 representative PHP applications, totaling over 10 million lines of code, as shown in Table 1. These applications were chosen based on three criteria: (1) Popularity, quantified by the number of GitHub stars, with a threshold of more than 1000 stars at the time of collection; (2) functional diversity, ensured by categorizing applications into different usage domains (e.g., content management systems, e-commerce, enterprise management, customer relationship management, project management), as summarized in the “Usage” column of Table 1; and (3) relevance to related work, focusing on applications that have been used in prior studies.

### 4.3. Procedure

**Figure 7:**
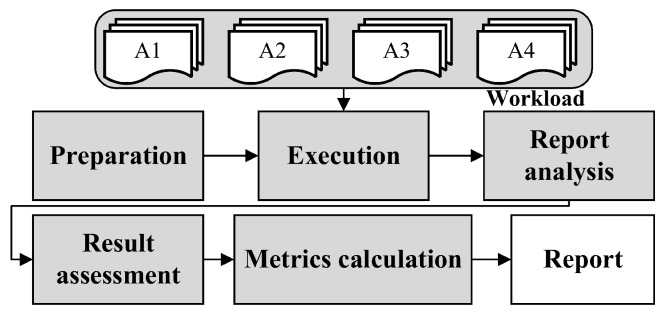
Benchmarking procedure.

The benchmarking procedure shown in Figure 7, is generally divided into five steps.

The first step is preparation, to determine and configure the SAST tool for benchmarking. The second step is execution, run the SAST tool, and perform benchmarking in the order of A1–A4. The third step is to report analysis, as the report styles of each SAST tool are not consistent. We need to extract key information from each SAST tool report and analyze the results according to the requirements of our metrics. The fourth step is result assessment, analyzing the statistics obtained in the third step, and evaluating the capabilities according to different workloads A1–A4. For example, in A2, combined verification is required, which cannot be characterized by the results obtained from a single test case. The fifth step is to calculate the metrics for each SAST tool based on the metrics. A more detailed implementation guide covering dataset setup, tool installation, and testing procedures will be published in our GitHub repository.

## 5. Results

In this section, we evaluate the vulnerability detection capability of the current state-of-the-art SAST tool (including RIPS [21], WHIP [30], Progpilot [31], WAP [32], PhpSafe [20], Tchecker [22], and Pixy [33]) from four key dimensions (A1–A4), with the main objective of demonstrating that our assessment system can progressively and comprehensively assess the vulnerability detection capability of SAST tools while explicitly modeling and controlling evaluation entropy to reduce analysis uncertainty. Among them, the evaluation of A2–A4 has already been completed in our previous work [23]. In this section, an evaluation experiment with the A1 dimension has been added, and its experimental environment setting is consistent with previous work, ensuring the effective integration and mutual reference of previous experimental conclusions and A1 dimension experimental results. It must be emphasized that previous research work is the starting point of this paper. Based on this, we further explore how to effectively and comprehensively evaluate the capabilities of PHP SAST tools and construct a targeted capability assessment system accordingly.

For the evaluation of the A1 dimension, as mentioned earlier, we conducted an in-depth investigation of all built-in functions in PHP [29], classified the built-in functions related to source, sanitation, and sink based on expert experience, as shown in Table 2.

For the Sink, we have generated a total of 104 test cases, including six types of vulnerabilities: Command Execution and Code Execution (CE), Sensitive Data Exposure (SDE), Cross-Site Scripting (XSS), Unrestricted File Upload (UFU), SQL Injection (SQLI), and File Manipulation (FM, including file inclusion and file deletion), as illustrated in Table 3. Among them, for the multi-parameter sink function, we generated negative test cases using non-hazardous parameter positions, that is, the part of the table using superscript M, while the single parameter sink function uses superscript S in the table.

In this dataset, Source samples include only positive examples, as Source is primarily considered the starting point for potential risks in security analysis. Negative examples for Source have no practical significance, as the concept of a “secure source” does not exist. In contrast, sanitization samples consist exclusively of negative examples, reflecting the primary function of sanitization as a mechanism to mitigate vulnerabilities or filter malicious inputs. Positive examples for sanitization are not included, as issues such as improper implementation or bypassing of sanitization are better analyzed as characteristics of re-sanitization or sink nodes, rather than being defined as positive examples for sanitization. For Sink, the risk associated with multi-parameter sink functions depends on which parameter receives the tainted data. For instance, if the first parameter is controllable, it may result in a vulnerability, whereas if the second parameter is controllable, it may not. These multi-parameter sink functions can generate both positive and negative examples, capturing the impact of different parameter positions on security. On the other hand, single-parameter sink functions typically produce only positive examples, as the controllability of a single parameter directly determines whether a vulnerability exists, leaving no room for negative examples. This design logic directly results in more positive examples than negative examples in the Sink portion of the dataset.

The evaluation results of A1 are shown in Table 4, demonstrating the identification ability of each tool for the three elements of taint analysis. For Source, almost all tools support the recognition of five source points, except for Progpilot and Tchecker, which do not support the recognition of $_FILE.

For the identification of sanitization, the performance of all tools is catastrophic, with FPR exceeding 80%, primarily because current SAST tools lack sufficient modeling of sanitization functions. Many functions that inherently disrupt taint semantics (e.g., mathematical functions such as acos) are not recognized as sanitization by the tools. We take the test case composed of the acos function as an example for the discussion, as shown in Listing 7. The acos function returns the arc cosine of num in radians. Obviously, even if the input of this function can be controlled, the result it returns is always a constrained numeric value that cannot realistically cause PHP application vulnerabilities. Since current PHP SAST tools generally only model traditional sanitization functions such as md5, this omission leads to a large number of false positives, resulting in FPR above 80%. Such high FPR introduces significant evaluation entropy and produces a large amount of erroneous taint propagation data streams in SAST tools.

**Listing 7.** A test case of sanitization assessment.

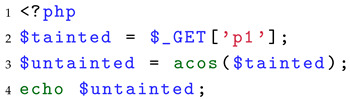



For Re-Sanitization, as mentioned earlier in Listing 4, Negative only uses test cases with encryption or encoding functions, while Positive uses the corresponding decryption or decoding functions in Negative test cases to correctly restore taint. From the experimental results, RIPS achieved 50 BAS, indicating that it successfully modeled some reversible sanitization functions, while others exceeded its modeling capabilities. For Pixy, which achieved 20 BAS, further analysis of its experimental results revealed that FP were lower than TP because certain functions used in the test cases were from higher versions of PHP, beyond Pixy’s capabilities, leading to runtime crashes. For other tools, most recorded 0 BAS, indicating their failure to correctly model these reversible sanitization functions and their inability to reduce entropy in reversible data transformations, which leads to uncontrolled uncertainty propagation. It indicates that these SAST tools did not model these reversible sanitization functions correctly, but used an incorrect assumption [34] that unmodeled functions can propagate taint.

**Figure 8:**
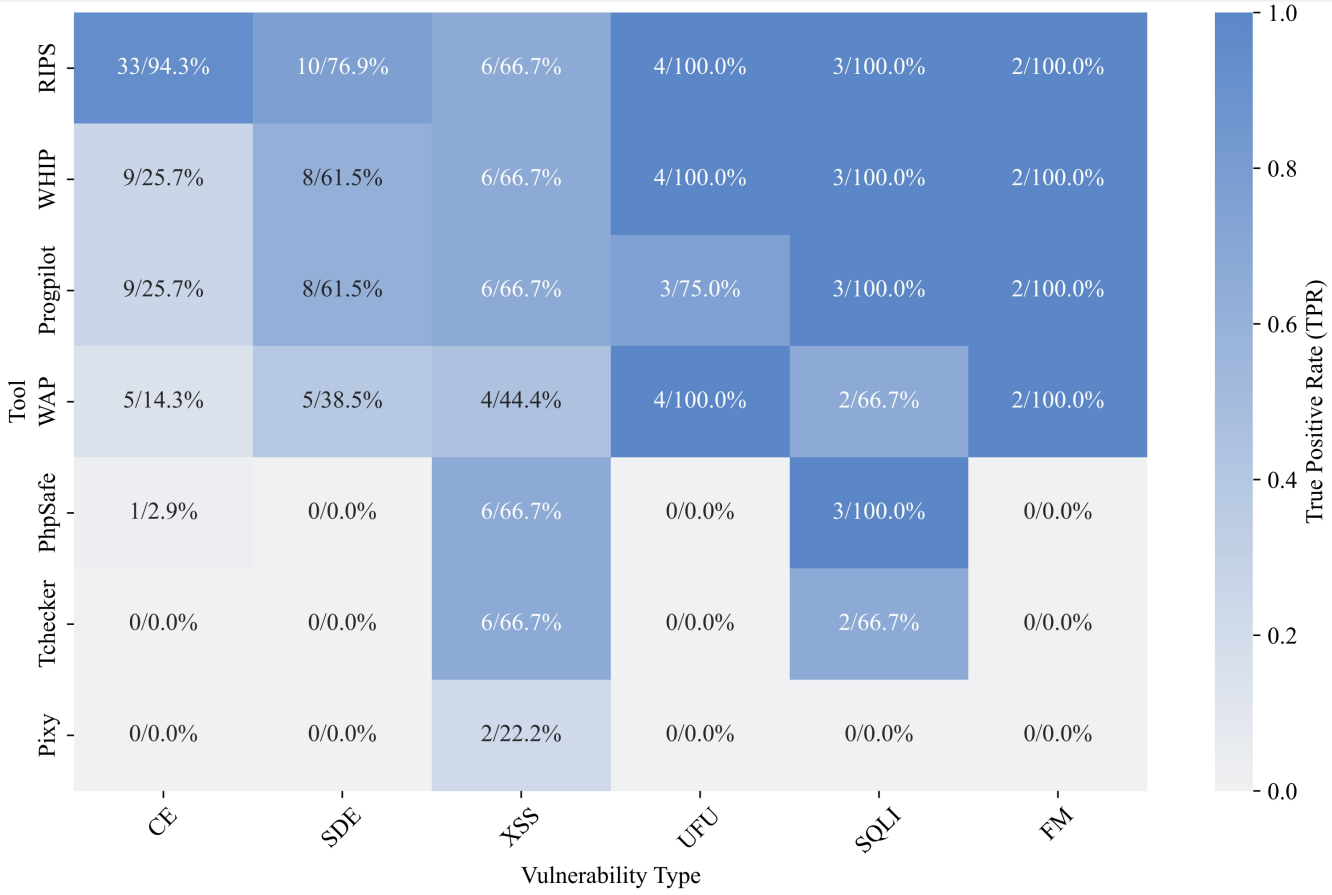
The evaluation results of positive sink test cases.

For Sink, overall, RIPS performed the best, achieving a TPR of 87.9%, while WHIP and Progpilot also achieved a TPR of over 45%. For other tools, the overall data performance is poor due to the uneven number of test cases for each vulnerability type in the test cases. From the perspective of BAS, RIPS also performed best, 72.1 BAS highlighting its ability to maintain a high true positive detection rate while effectively controlling false positives. Next, we further analyze the performance of these tools in different types of vulnerabilities. Figure 8 shows the evaluation results of all tools for positive test cases in Sink.

For the types of vulnerabilities that can be detected, Pixy can only detect XSS vulnerabilities, Tchecker can detect XSS and SQLI vulnerabilities, Phpsafe further supports the detection of CE vulnerabilities, and other tools support six types of vulnerabilities in test cases. Although Tchecker and Phpsafe do not support many types of vulnerabilities, they perform the best or second best in the sink function evaluation of XSS and SQLI.

For the remaining four tools, the gap is widened by their support for CE vulnerability type detection. RIPS supports the detection of 94.3% of sink functions in test cases, while the second-best WHIP only has 25.7% TPR. For other types of vulnerabilities, these four tools perform well, especially in terms of support for UFU, SQLI, and FM vulnerability types, almost all achieving 100% support.

**Figure 9:**
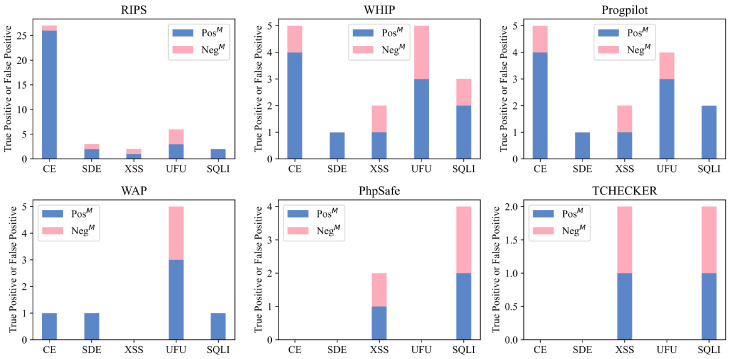
The evaluation results of multi-parameter sink test cases.

Next, we further analyze the multi-parameter sink function recognition capability in sink recognition. The experimental results are shown in Figure 9.

As mentioned earlier in Listing 5, the negative test cases in the multi-parameter sink function set the taint passing to a nondangerous parameter position, which does not cause vulnerabilities, while the positive test cases set the taint to a dangerous parameter position. It is worth mentioning that because there is no multi-parameter sink function in the FM type, and RIPS does not report any warning for any multi-parameter sink function test cases, the results of RIPS and FM have been excluded from the figure. In the experimental results, PhpSafe and Tchecker reported the same proportion of positive and negative examples for XSS and SQLI type multi-parameter sink functions, indicating that they did not distinguish the parameter positions of multi-parameter sink functions and could not accurately identify multi-parameter sink functions. For other tools, WAP only reports warnings for Negative test cases in the detection of UFU-type vulnerabilities. It has good recognition ability for multi-parameter sink functions, but its sink function modeling quantity is relatively small. RIPS reported some warnings for Negative test cases, but overall its ability to recognize multi-parameter sink functions is good. WHIP and Progpilot need to further increase the number of sink function models and focus on identifying multi-parameter sink functions.

The evaluation results of the A1 dimension show that the current state-of-the-art tool performs poorly in the recognition capability of the most basic sanitization and sink functions. On the one hand, unrecognized sanitization functions are mistakenly regarded as taint propagation functions, leading to over-taint and an increase in tool false positive rates; On the other hand, the limited number of identifiable sink functions makes it impossible to raise an alarm even if the taint reaches the sink function, increasing the tool’s false negative rate.

The evaluation results of A2–A4 dimensions were completed in our previous work [23], and the main conclusions drawn are as follows: (1) The current state-of-the-art tool does not have the basic data flow analysis capability to meet the standards, and only reflects a part of the flow-sensitive analysis and interprocedural analysis capability in the evaluation. (2) The current state-of-the-art tools do not have sufficiently complex semantic analysis, and there is still a lot of room for improvement in type inference of arrays and objects, analysis of dynamic characteristics, and modeling of built-in functions. (3) The current state-of-the-art tools can detect some real-world vulnerabilities, but the extremely high false positive rate introduces overwhelming information noise and evaluation entropy, leading to real-world vulnerabilities being masked by massive false positive information, which seriously restricts the practical use of SAST tools in production environments.

In summary, when a PHP SAST tool struggles with A1 and A2 dimensions, its performance in the A3 dimension is significantly affected. Type inference heavily relies on the basic data flow analysis ability (A2), while analyzing dynamic features depends on both the accuracy of type inference and data flow analysis (A2). Additionally, analyzing built-in functions depends on sink function recognition (A1), basic data flow analysis (A2), and type inference ability. The ultimate assessment of real vulnerability detection capability (A4) is clearly dependent on the capabilities of A1–A3. If a PHP SAST tool falls short in A1–A3, its performance in detecting real vulnerabilities will inevitably suffer. These four dimensions form a progressive, entropy-aware assessment system that gradually reduces analysis uncertainty and highlights, revealing deficiencies and shortcomings in PHP application vulnerability detection from multiple perspectives. Compared to current benchmark testing methods, more comprehensive evaluation metrics have been proposed, offering specific guidance for future improvements of PHP SAST tools.

## 6. Discussion

This section describes the current limitations of our method and our plans to address these limitations in future work, and it provides improvement suggestions for the SAST tools based on the evaluation results.

In-depth evaluation of basic data flow analysis capabilities. At present, our evaluation methods for basic data flow capabilities only involve the most basic branch. When SAST tools can pass the evaluation of these test cases, it is difficult to further distinguish the gap in the data flow analysis capabilities of these tools. We plan to further research and design more complex data flow analysis test cases.

Considering that object-related analysis is currently a challenge for PHP SAST tools, we did not include an assessment of the recognition ability of sanitization and sink functions related to objects in the A1 dimension. In future work, we consider placing this part after the A3 dimension and evaluating it when the PHP SAST tool has certain object type inference capabilities.

Automated analysis of new features and functions. Currently, the test cases used in our evaluation method mainly rely on in-depth analysis of PHP documentation by expert experience. When PHP has new features or built-in function updates, it is not possible to automatically analyze and generate corresponding test cases to improve our benchmark. We plan to combine natural language processing and large language model-related methods to achieve automated updating and iteration of benchmarks.

Statistical analysis of real-world application vulnerabilities. Our evaluation of the vulnerability detection capability of SAST tools mainly relies on horizontal comparisons of the number of detections and the uniqueness of detected vulnerabilities, and cannot further analyze the preference of different SAST tools for vulnerability detection. We plan to study a vulnerability feature statistical algorithm to determine which types of vulnerabilities different SAST tools are more adept at detecting. These features may include how many built-in functions are used on the taint propagation path of the vulnerability, whether object handling is involved, which branches are involved, and so on.

Regarding the selection of tools, we chose those that are widely recognized as the most advanced methodologies in the field, while there may be similarities in the architecture and design of these tools, we believe this reflects a significant limitation in the current research landscape: the lack of a standardized benchmark dataset. This absence makes it challenging for researchers to conduct comprehensive and consistent evaluations of tool effectiveness, leading to designs that tend to revolve around specific assumptions or functionalities.

Based on the evaluation results, to improve the effectiveness of static analysis for web applications, we recommend that SAST tools consider adopting PHP opcodes as an abstraction for program semantics. This can help minimize semantic entropy introduced by AST-based control flow modeling, which in practice often involves additional complexity and may lead to inaccuracies. Unlike abstract syntax trees (ASTs) [35], which primarily capture code structure, PHP opcodes [36] provide a lower-level and more explicit representation of control flow, thereby supporting more accurate construction of control flow graphs (CFGs). This observation, validated in our prior work [23], suggests that opcode-based abstractions can facilitate precise static analysis algorithms and improve the handling of complex PHP applications.

## 7. Conclusions

In this paper, we have developed a systematic, entropy-aware evaluation framework for PHP SAST tools, designed to provide accurate and comprehensive assessments of their vulnerability detection capabilities. The framework explicitly models and controls evaluation entropy and focuses on isolating key factors affecting data flow analysis. It evaluates tool performance across the following four progressive dimensions: the capability to identify the three elements of taint analysis, basic data flow analysis, complex semantic analysis in PHP applications, and accurately identifying vulnerabilities in real-world applications. By reducing uncontrolled variability and quantifying entropy reduction in each assessment layer, the framework enables the more reliable measurement of detection capabilities and highlights where uncertainty accumulates in current SAST tools. Using a specific benchmarking instance, we validated the feasibility of the framework, and the experimental results demonstrated its ability to effectively identify limitations in current SAST tools and provide clear directions for their future improvement.

## Figures and Tables

**Table 1:** Overview of representative list of the applications used.

#	Application	Version	Stars	LoC	Usage	#	Application	Version	Stars	LoC	Usage
1	wordpress	6.6	19.1k	585,422	Content Management Systems	13	openemr	6_0_0	2.9k	844,467	Healthcare Management
2	DVWA	1.9	9.7k	33,884	Vulnerability Learning	14	shopware	6.4.4.0	2.7k	581,328	Business Platforms
3	dolibarr	12.0.0	5.1k	63,445	Enterprise Management	15	phpipam	1.6	2.2k	194,546	IP Address Management
4	organizr	1.9	5k	1,331,842	Server Management	16	WDScanner	latest	2k	6692	Network Scanning
5	Joomla	5.1.2	4.7k	979,753	Content Management Systems	17	bjyadmin	latest	1.8k	288,839	ThinkPHP Management
6	SuiteCRM	7.12.6	4.3k	968,371	Customer Relationship Management	18	Gazelle	latest	1.8k	85,026	BitTorrent Tracking
7	leantime	2.1.5	4.3k	1,492,972	Project Management	19	phpbb	3.3.10	1.8k	355,350	Content Management Systems
8	glpi	10.0.16	4k	36,930	Asset Management	20	unmark	1.9.2	1.6k	83,409	Bookmark Management
9	dzzoffice	2.02.1	3.9k	196,872	Office Suites	21	icecoder	8.1	1.4k	14,040	Code Editors
10	librenms	21.1.0	3.6k	258,484	Network Monitoring	22	openflights	latest	1.4k	12,049	Flight Data Storage
11	microweber	2.0.16	3.1k	409,969	E-commerce	23	RPI-Jukebox	2.7.0	1.3k	10,128	Jukebox
12	microweber	1.2.3	3.1k	291,880	E-commerce	24	PicUploader	latest	1.2k	1,516,425	Image Hosting Tools

**Table 2:** Overview of Dataset for A1.

Type	Pos	Neg	Total
Source	5	0	5
Sanitization	0	861	861
Re-Sanitization	20	20	40
Sink	66	38	104
Sum	91	919	1010

**Table 3:** Sink Details in Dataset for A1.

Type	Pos^*M*^	Neg^*M*^	Pos^*S*^	Total
CE	28	28	7	63
SDE	3	3	10	16
XSS	2	2	7	11
UFU	3	3	1	7
SQLI	2	2	1	5
FM	0	0	2	2
Sum	38	38	28	104

**Table 4:** Overview of the A1 Evaluation.

	Source	Sanitization	Re-Sanitization			Sink		
	TP (TPR)	FP (FPR)	TP (TPR)	FP (FPR)	BAS	TP (TPR)	FP (FPR)	BAS
RIPS	5 (100.0%)	691 (80.3%)	19 (95.0%)	9 (45.0%)	50	58 (87.9%)	6 (15.8%)	72.1
WHIP	5 (100.0%)	758 (88.0%)	19 (95.0%)	18 (90.0%)	0	32 (48.5%)	5 (13.2%)	35.3
Progpilot	4 (80.0%)	704 (81.8%)	9 (45.0%)	9 (45.0%)	0	31 (47.0%)	3 (7.9%)	39.1
WAP	5 (100.0%)	735 (85.4%)	18 (90.0%)	18 (90.0%)	0	22 (33.3%)	3 (7.9%)	25.4
PhpSafe	5 (100.0%)	701 (81.4%)	13 (65.0%)	14 (70.0%)	−5	10 (15.2%)	3 (7.9%)	7.3
TCHECKER	4 (80.0%)	730 (84.8%)	18 (90.0%)	18 (90.0%)	0	8 (12.1%)	2 (5.3%)	6.8
Pixy	5 (100.0%)	819 (95.1%)	19 (95.0%)	15 (75.0%)	20	2 (3.0%)	0 (0.0%)	3

## Data Availability

The datasets generated during the current study are publicly available in the PSAbench repository, https://github.com/xjzzzxx/PSAbench.
